# The Global Youth Tobacco Survey (GYTS): linking data to the implementation of the WHO Framework Convention on Tobacco Control

**DOI:** 10.1186/1471-2458-8-S1-S1

**Published:** 2008-12-15

**Authors:** Charles W Warren

**Affiliations:** 1 Centers for Disease Control and Prevention, Office on Smoking and Health, Global Tobacco Control Program, 3005 Chamblee Tucker Road, Atlanta, GA 30341, USA

## 

The World Health Organization (WHO), the US Centers for Disease Control and Prevention, and the Canadian Public Health Association developed the Global Tobacco Surveillance System (GTSS) to assist WHO member states in establishing continuous tobacco control surveillance and monitoring. The GTSS provides a flexible system that includes common data items but allows countries to include important unique information at their discretion. It also uses a common survey methodology, similar field procedures for data collection, and similar data management and processing techniques. The GTSS includes collection of data through three surveys: the Global Youth Tobacco Survey (GYTS) for youth, and the Global School Personnel Survey and the Global Health Professional Student Survey for adults.

Countries can use data from the GYTS to monitor and evaluate National Tobacco Control Action Plans and articles from the WHO Framework Convention on Tobacco Control (WHO FCTC). The WHO FCTC was adopted by the 56th World Health Assembly in May 2003 and became international law on February 27, 2005. It is the world's first public health treaty on tobacco control and encourages countries that ratify to develop and implement tobacco control policies, such as bans on direct and indirect tobacco advertising, tobacco tax and price increases, promoting of smoke-free public places and workplaces, and placement of health messages on tobacco packaging.

The WHO FCTC, National Tobacco Control Action Plans, and the GYTS share the same goal: the development, implementation, and evaluation of effective tobacco control programs in all WHO member states. What the WHO FCTC and National Tobacco Control Action Plans ask countries to monitor, the GYTS can help to measure. The GYTS provides indicators for measuring achievement of several WHO FCTC articles (surveillance and monitoring, prevalence, exposure to secondhand smoke, school-based tobacco control, cessation, media and advertising, and minor's access and availability).

The synergy between the WHO FCTC, National Tobacco Control Action Plans, and GYTS offers countries a unique opportunity to develop, implement, and evaluate their comprehensive tobacco control programs. The reports included in this publication from Peru, Thailand, and Turkey are presented as examples of 'linking' GYTS data to the national tobacco control program efforts in each country.

## List of abbreviations used

FCTC: Framework Convention on Tobacco Control; GTSS: Global Tobacco Surveillance System; GYTS: Global Youth Tobacco Survey; WHO: World Health Organization.

## Competing interests

The author declares that they have no competing interests.

